# Vascular response and silent micro-embolization after rotational atherectomy and drug-coated balloon angioplasty: insights from micro-computed tomography and histology

**DOI:** 10.1093/ehjci/jeag117

**Published:** 2026-05-13

**Authors:** Kohei Hirobe, Sho Torii, Yu Sato, Sawako Chiba, Fumiaki Mori

**Affiliations:** Department of Cardiology, National Hospital Organization Yokohama Medical Center, 3-60-2 Harajuku, Totsuka-ku, Yokohama, Kanagawa 245-8575, Japan; Department of Cardiology, Tokai University School of Medicine, 143 Shimokasuya, Isehara, Kanagawa 259-1193, Japan; Department of Cardiology, Tokai University School of Medicine, 143 Shimokasuya, Isehara, Kanagawa 259-1193, Japan; Department of Pathology, National Hospital Organization Yokohama Medical Center, 3-60-2 Harajuku, Totsuka-ku, Yokohama, Kanagawa 245-8575, Japan; Department of Cardiology, National Hospital Organization Yokohama Medical Center, 3-60-2 Harajuku, Totsuka-ku, Yokohama, Kanagawa 245-8575, Japan

## Case description

A 72-year-old man with ischaemic cardiomyopathy underwent revascularization for three-vessel coronary disease under intra-aortic balloon pump support. Baseline coronary angiography showed bulky calcification extending from the left main trunk into the proximal left anterior descending artery (LAD; *Panel A*). Intravascular ultrasound after 1.5-mm rotational atherectomy (RA) demonstrated thick eccentric calcification with reverberation artefacts (*Panel C*). The lesion was then dilated with a non-slip element balloon and finalized with a drug-coated balloon (DCB).

Although the final angiogram showed adequate lumen gain without slow flow (*Panel B*), the patient developed post-procedural ST-segment elevation and progressive heart failure, dying 1 month later.

Post-mortem coronary histology (*Panels D* and *E*) and micro-computed tomography (micro-CT; *Panel F*; see Supplementary data online, *Movie S1*) showed effective modification of sheet-like and nodular calcification, with a smooth luminal surface and minimal injury to the non-calcified vessel wall. Notably, the absence of significant medial tears, an established pathological trigger for restenosis in calcified lesions, may have contributed to the excellent short-term patency with DCB-only therapy.

Myocardial micro-CT identified approximately 13 calcific fragments (yellow dotted circles) in the LAD territory across multiple sections, consistent with distal micro-embolization (*Panels G* and *H*, a magnified view of the white dotted box in *Panel G*). Myocardial histopathology demonstrated calcific material (arrow) within distal myocardial capillaries, accompanied by phagocytosing macrophages (arrowheads, *Panel I*).

This case provides first-in-human imaging and pathological evidence of angiographically ‘silent’ distal embolization after RA-assisted DCB. These observations support a conservative atherectomy approach and heightened vigilance for distal embolic events, particularly in vulnerable patients.

**Figure:**
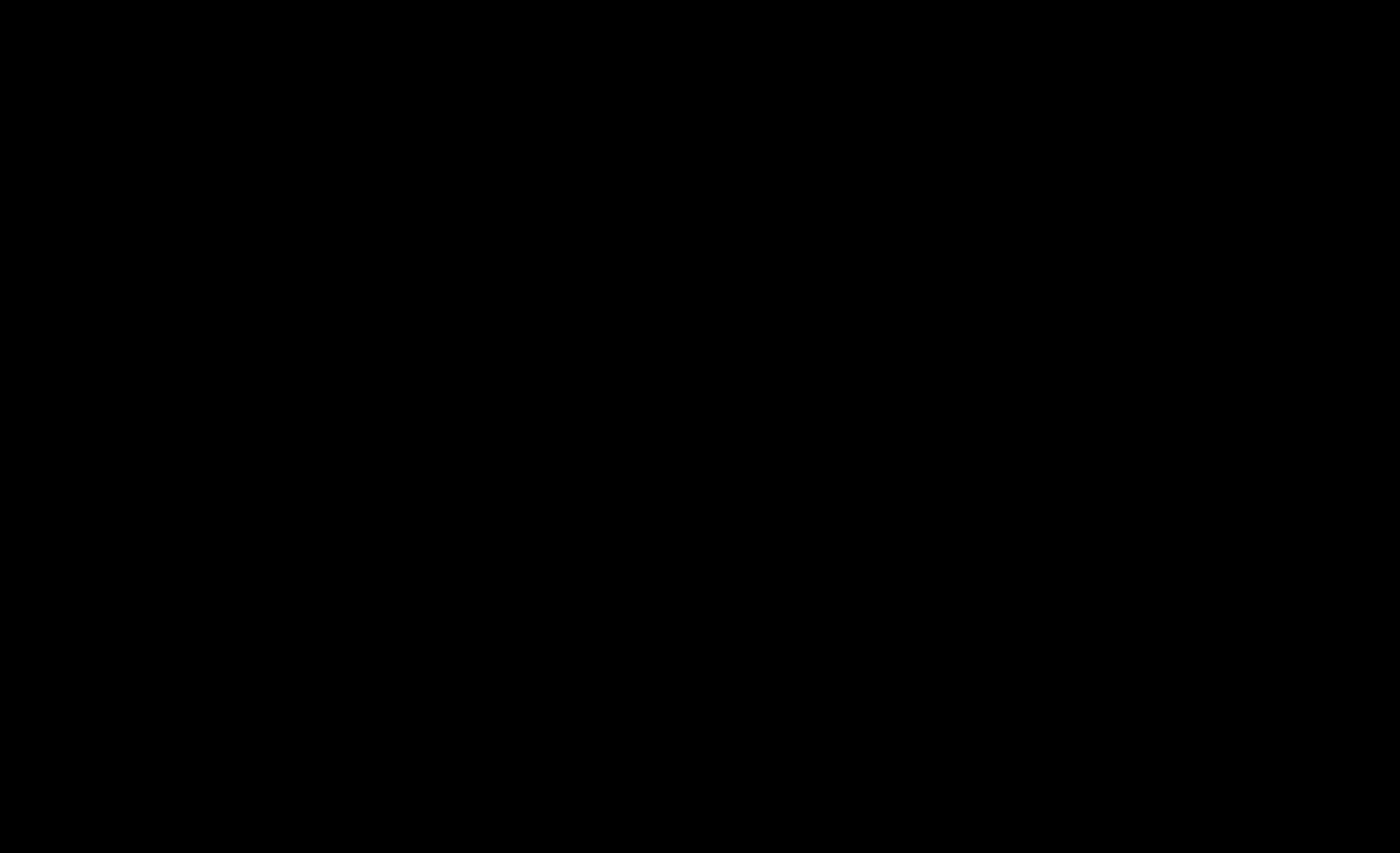


## Supplementary Material

jeag117_Supplementary_Data

## Notes

**Author contributions:** Kohei Hirobe (Writing—original draft [lead]), Sho Torii (Writing—review & editing [lead]), Yu Sato (Writing—review & editing [supporting]), Sawako Chiba (Formal analysis [supporting]), and Fumiaki Mori (Writing—review & editing [supporting])

**Statement of consent:** The authors confirm that written consent for submission and publication of this case report including images and associated text has been obtained from the patient in line with COPE guidance.

**Funding:** No financial support was provided for this manuscript. All authors have no relationships relevant to the contents of this paper to disclose.

**Data availability:** The data underlying this article are available within the article. Additional de-identified data will be shared by the corresponding author on reasonable request.

